# Isolated Growth Hormone Deficiency IA due to a Novel Homozygous Large Deletion ∼1.6 kb Spanning Exons 1–4 of GH1 Gene: A Case Report

**DOI:** 10.1002/ccr3.70234

**Published:** 2025-02-19

**Authors:** Shahab Noorian, Hedieh Soltani, Fatemeh Aghamahdi, Shahram Savad, Mahnaz Seifi Alan

**Affiliations:** ^1^ Department of Pediatric Endocrinology, School of Medicine Alborz University of Medical Sciences Karaj Iran; ^2^ Student Research Committee, School of Medicine Alborz University of Medical Sciences Karaj Iran; ^3^ Department of Medical Genetics, School of Medicine Tehran University of Medical Sciences (TUMS) Tehran Iran; ^4^ Cardiovascular Research Center Alborz University of Medical Sciences Karaj Iran

**Keywords:** case report, GH1 gene deletion, growth hormone deficiency, isolated growth hormone deficiency, short stature, WES

## Abstract

Isolated growth hormone deficiency (IGHD) IA is inherited autosomal recessively and occurs due to GH1 gene deletions. This study emphasizes the importance of clinical diagnosis and molecular examination for detecting novel mutations to prevent misdiagnosis and to consider timely and appropriate management of the current and long‐term consequences of the defect, such as additional deficiencies. We report a male infant who initially presented with a growth delay (‐4SD) at 4 months old, and at 16 months old, was referred to our endocrinology department. Physical examination revealed developmental delay, macrocephaly, head lag, loose body, large head circumference, low and flat nasal bridge, forehead protuberance, three‐pronged fingers, shortness of the hands and feet joints, small palms and plantar, small penises, delayed tooth eruption, and disability to walk. His hormonal tests showed normal free T4 (14.08 pmol/L), upper limit TSH level (5.41 μIU/mL), normal random cortisol 8 AM (250.53 μg/mL), mild high ACTH level (79.6 pg/mL), low IGF1 (13.0) and fasting GH (0.03 ng/mL). GH (ng/mL) maximal response to the arginine test was < 0.05. Whole Exome Sequencing, Polymerase Chain Reaction (PCR), and Quantitative Real‐Time PCR were performed on a peripheral blood sample obtained from the patient. The infant was found to have a homozygous large deletion of approximately 1.6 kb spanning the GH1 gene, which contained exons 1–4 with an autosomal recessive inheritance and a heterozygous deletion of exons 1–4 of GH1 in the parents and homozygous wild‐type in the sibling. Novel mutations in the GH‐1 gene cluster are considered an important cause of idiopathic congenital IGHD. As a result, the role of gene sequencing, besides considering clinical features, should not be neglected.

**Summary**

- Isolated growth hormone deficiency can have different inheritance patterns depending on the type of the condition. Isolated growth hormone deficiency types IA and IB are inherited in an autosomal recessive pattern, which means both copies of the GH1 or GHRHR gene in each cell have mutations.
- Isolated growth hormone deficiency is caused by mutations in one of at least three genes. Isolated growth hormone deficiency types IA and II are caused by mutations in the GH1 gene. Type IB is caused by mutations in either the GH1 or GHRHR gene. Type III is caused by mutations in the BTK gene.

## 1 Introduction

Isolated growth hormone deficiency (IGHD) can occur due to congenital or acquired mutations affecting the GH's production and release. Mutations in the genes that encode growth hormone (*GH1*), growth‐hormone‐releasing hormone receptor (*GHRHR*), and transcription factor *SOX3*, *with variations in PROP1*, *POU1F1*, *LHX3*, *LHX4*, *SOX2*, *STAT5B*, *IGF1*, *IGFALS*, *GLI2*, and *HESX1*, are the most common causes of IGHD [1, 2, 3]. IGHD can be familial or sporadic. Most cases are sporadic, whereas familial ones are rare and divided into four categories based on inheritance and Mendelian patterns [1, 4, 5]. These patterns include IGHD types IA (absence of endogenous GH) and IB (decreased GH), which are inherited autosomal recessively, IGHD type II with autosomal dominant inheritance (most common), and IGHD III in an X‐linked manner. The *GH‐1* gene is located on the long arm of chromosome 17 (17q22‐24). Deletions in the *GH‐1* gene cause IGHD IA, and the most common related reason is unequal and inadequate crossover and recombination during the meiosis of the GH gene region [6]. Patients clinically present with short stature and severe growth delay during the first 6 months of life (with a height SDS score less than −4.50 SD). However, they might exhibit normal body length and weight at birth, especially in type IA [7].

This study describes a case of IGHD presented with growth retardation (−4SD) after birth and revealed developmental delay, macrocephaly, delayed neck development, loose body, large head circumference, low and flat nasal bridge, forehead protuberance, three‐pronged fingers, shortness of the hands and feets joints, small palms and plantar, small penises, delayed tooth eruption, and inability to walk, due to a novel homozygous large deletion of approximately 1.6 kb spanning the GH1 gene in order to emphasize the importance of clinical diagnosis and molecular examination for detecting novel mutations to prevent misdiagnosis and develop timely and appropriate treatment, besides considering the side effects of this defect if it remains untreated.

## 2 Case Presentation/Examination

### 2.1 General Data

**FIGURE 1:**
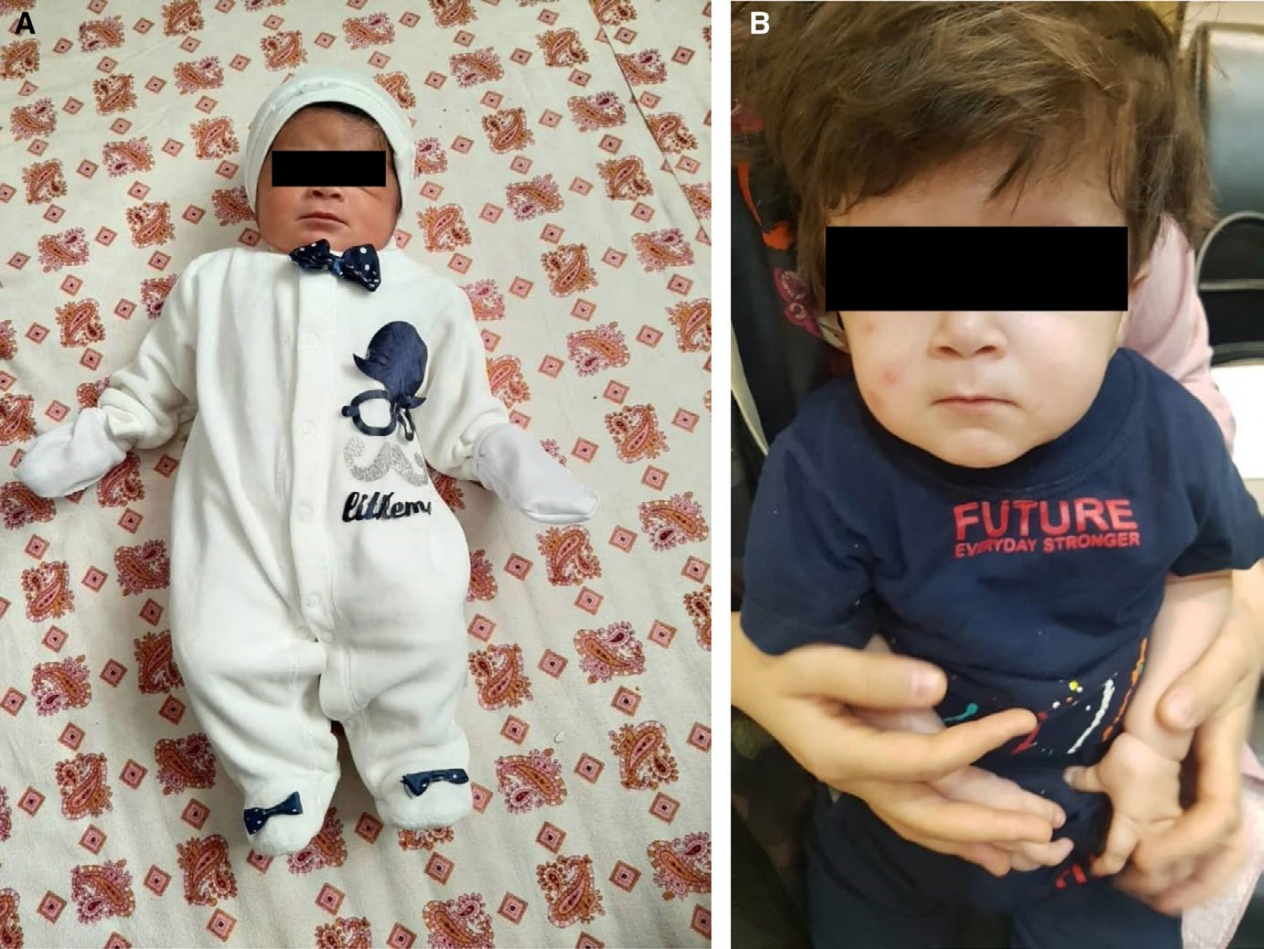
Patient at 1 and 12 months' old.

We report a 16‐month‐old male born with a birth weight of 3.5 kg, a length of 49 cm, and a head circumference of about 37 cm (Figure 1).

His parents were first cousins, and he was their second child. They did not mention any history of related diseases in the family, and their first child was entirely normal. He was born by vaginal delivery after 38 weeks and 5 days. His mother had a stressful pregnancy with poor nutrition, and in the third trimester, she was on complete bed rest. He was completely breastfed until 1 year old. His father was 172 cm, and his mother was 159 cm.

### 2.2 Physical Examination

Loose body, delayed neck development, large head circumference, and developmental delay at 4 months old were the first manifestations that his mother noticed. At 16 months old, the infant was referred to the endocrine unit of our pediatric hospital due to growth delay. At the time of the visit, his height was 67 cm (−4 SD), his weight was 6 kg, and his head circumference was about 43 cm (Table 1). Specific clinical characteristics on physical examination included developmental delay, macrocephaly, low and flat nasal bridge, forehead protuberance, three‐pronged fingers, shortness of the hands and feets joints, small palms and plantar, and head lag, as we call flappy baby. Micro penis was obvious in him. His abdomen was soft without tenderness, guarding, or distension; His spleen and liver were of normal size and palpable.

**TABLE 1:** Growth chart.

Age (months)	0	4	12	24 (8 months after hGH replacement therapy)
Body length (cm)	49	67	67	73
Body weight (kg)	3/560	6	7/400	8
Head circumference (cm)	37	43	45	48/5
BMI	14.8	13.4	16.5	15

## 3 Methods

### 3.1 Medical Imaging

A hip ultrasound was performed at 1 month old, which is summarized in Table 2, and No evidence of DDH was observed in both hips. A brain transfontanelle ultrasound performed at 5 and 6 months old reported normal structures. At 10th months old, a brain MRI was accomplished and reported prominence of the ventricle and extra‐axial CSF space in favor of benign hydrocephaly of infancy (Table 3).

**TABLE 2:** Hip ultrasound.

	Bony roof (angle A)	Cartilage roof (angle B)	Age (week)	Hip type
RT. Hip	66	41	5	1α
LT. Hip	68	39	5	1α

**TABLE 3:** Summary of the imaging studies.

Imaging study	Age (months)	Report
Hip ultrasound	1	No evidence of DDH
Brain Trans‐fontanelle ultrasound	5	Normal parenchyma at white and gray matter, normal midline ingredients, and no apparent midline shift Normal corpus callosum, IHF, gyrus, and sulci forms and no signs of ventriculomegaly or intraventricular hemorrhage The size and shape of the ventricles were acceptable
Brain ultrasound	6	Same results Normal CSF and cerebellum No evidence of IPH, IVH, and GMH
Brain MRI with Diffusion‐weighted and without contrast	10	Prominence of the ventricle and extra‐axial CSF space in favor of benign hydrocephaly of infancy, unremarkable bilateral thalami, basal ganglia, pituitary, orbit, and optic nerve, posterior fossa, CPA, IACs, and no evidence of gross structural abnormality, mass effect, ICH and obvious restriction on DWT sequence Normal myelination process according to the patient's age, and white/gray matter signals were within normal limits Sellar and supra‐sellar structures had normal appearances and signals

### 3.2 Laboratory Assessment

Renal, hepatic, intestinal, or metabolic etiologies for growth failure were excluded due to normal serum electrolytes, kidney and liver function tests, blood gas analysis, and biochemical tests. The thyroid function indicators, including total T4 (86.0 nmol/L), free T4 (13.1 pmol/L), and TSH (1.47 μIU/mL), were in the normal range at 4 months old. His hormonal tests at 1 year and 10 months old revealed normal free T4 (14.08 pmol/L), upper limit TSH level (5.41 μIU/mL), and IGF1 (13.0 ng/mL). GH (ng/mL) maximal response to the arginine test was < 0.05. She also had normal random cortisol at 8 AM (250.53 ng/mL), with a mild high ACTH level (79.6 pg/mL). Both fasting GH (0.03 ng/mL) and IGF1 (13.0 ng/mL) levels were low (Table 4). The laboratory tests were measured by ELISA (for TSH), ECLIA (for free T4, Fasting GH), ELFA (for Cortisol Am and Free T4), and CLIA (for IGF 1 and ACTH).

**TABLE 4:** Laboratory findings.

Age (month)	4	Normal range	22	Normal range
TSH (μIU/mL)	1.47	0.73–8.35	5.41	0.7–5.97
Total T4 (nmol/L)	86.0	73–206		
Free T4 (pmol/L)	13.1	9–24	14.08	12–22
ACTH (pg/mL)			79.6	7–65
8 am Cortisol (ng/mL)			250.53	54.94–287.56
Fasting GH (ng/mL)			0.03	0.094–6.29
GH peak (ng/mL) after arginine			< 0.05	> 10
IGF 1 (ng/mL)			13.0	23.9–183.9

Abbreviations: ACTH, adrenocorticotropic hormone; GH, growth hormone; IGF 1, Insulin like growth factor 1; TSH, thyroid stimulating hormone.

### 3.3 Genetic Test

Genomic DNA was extracted from the blood sample and subjected to Whole Exome Sequencing (WES). The target regions were enriched using Agilent SureSelect V7, and an average depth of 100× coverage was obtained for over 98% of the targeted bases using the Illumine NovaSeq sequencer. The aligned paired‐end 150 base‐pair reads were compared to the human reference genome (GRCh37/hg19). The analytical sensitivity for single nucleotide variants and small insertions/deletions (< 5 bp) was estimated to be greater than 97%. Pathogenic and likely pathogenic variants were identified based on the ClinVar database, using the numbering and nomenclature recommended by the Human Genome Variation Society (HGVS, http://www.hgvs.org/). The variant classification was consistent with the standards and guidelines of the American College of Medical Genetics and Genomics (ACMG).

To confirm the deletion, we developed a primer pair (F: CTAAGGAGCTCAGGGTTTTTCC and R: GGAATGAATACTTCTGTTCCTTTGG) that specifically amplifies exon 3 of the GH1 gene, one of the deleted exons. Polymerase Chain Reaction (PCR) was conducted at an annealing temperature of 63°C, producing 169 bp amplicons that were used to confirm the deletion. The PCR products were subjected to electrophoresis on a 2% agarose gel, and the deletion was confirmed in the patient (Figure 1).

To determine the copy numbers of GH1 exon 3 in the patient and her family members (parents and siblings), we also employed a SYBR Green‐based real‐time PCR assay [8]. The assay involves amplification of the ALB (Albumin) gene as a reference gene on chromosome 4, as well as GH1 exon 3.

The real‐time PCR primer pairs consist of GH1 (as PCR primers) and ALB (F: AGCTATCCGTGGTCCTGAAC, R: TTCTCAGAAAGTGTGCATATATCTG) sequences. The reaction mixtures were carried out in a final reaction volume of 20 μL, which contained 10 μL of SYBR Green Master Mix, 1.25 μL of each primer (250 nM), 1 μL template DNA (50 ng), and 6.5 μL of water. The assay utilized genomic DNA from the patient and their family, as well as a healthy individual's genomic DNA as a reference.

The thermocycling conditions consisted of 5 min at 95°C, followed by 40 cycles of 15 s at 95°C and 60 s at 60°C. To ensure the specificity of the PCR reaction, a melting curve was generated for each PCR product after amplification. The copy numbers of GH1 exon 3 were calculated using the delta–delta CT method.

## 4 Result and Conclusion

**FIGURE 2:**
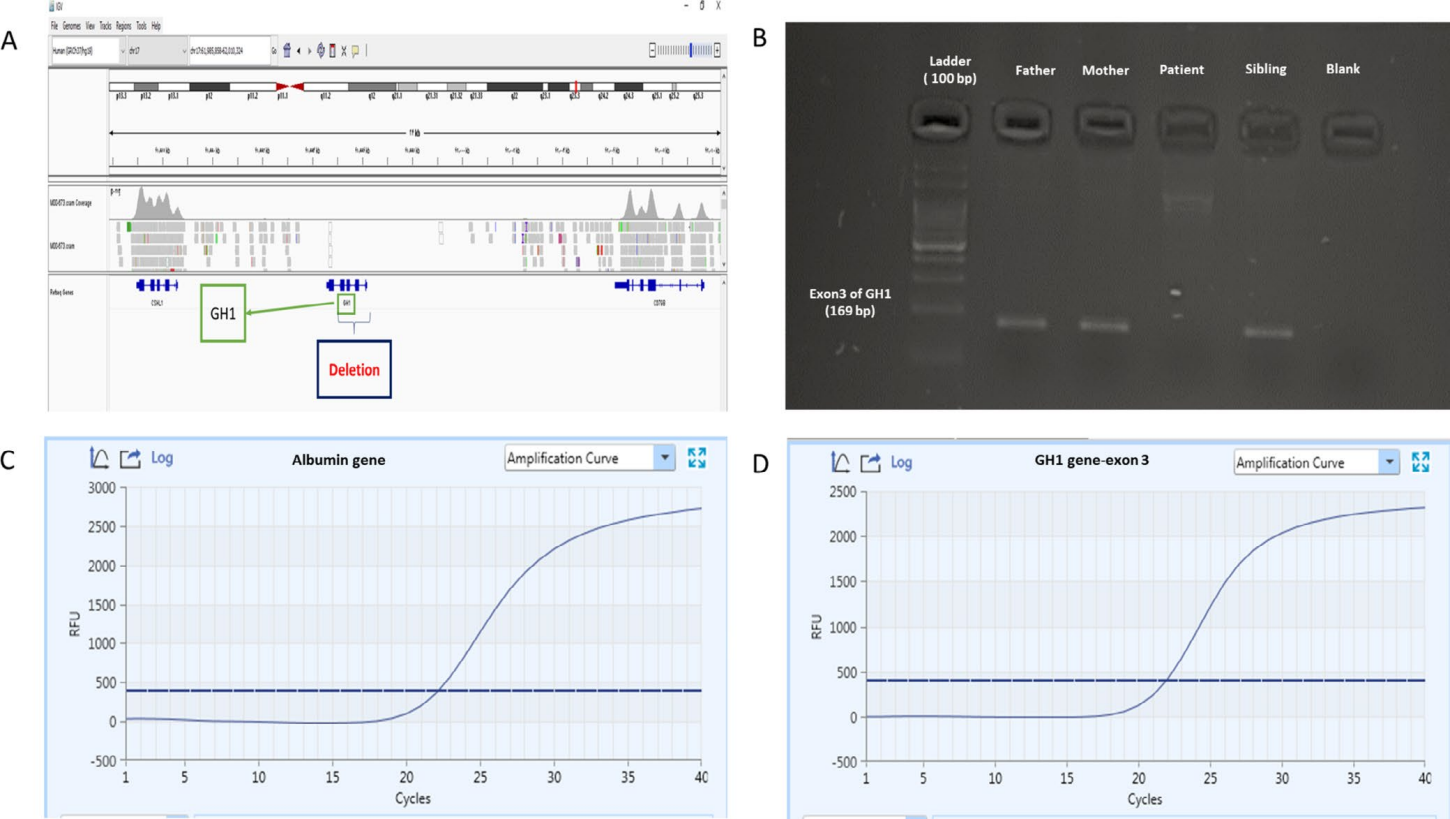
Summary of molecular genetic analyses. (A) Screenshot of the Integrative Genomics Viewer (IGV) window presenting the deletion encompassing exons 1–4 in the GH1 gene. (B) Results of the PCR‐electrophoresis analysis in the patient and healthy member of the family. (C&D) The Real‐Time PCR product amplification curve results for ALB and GH1 genes.

Due to severe short stature, low endogenous growth hormone levels, inappropriate response to the provoked test, and parental relativity, a genetic analysis using WES was performed on genomic DNA extracted from an EDTA‐treated peripheral blood sample. The analysis revealed a novel homozygous large deletion of about 1.6 kb spanning exons 1–4 of the GH1 gene on chromosome 17, causing autosomal recessive growth hormone deficiency. The Integrative Genomics Viewer (IGV) data is illustrated in Figure 2.

PCR and gel electrophoresis results showed no band for the patient, but the formation of a band for his family members confirmed the patient's homozygous deletion. Quantitative real‐time PCR was performed to determine the zygosity of the deletion in her family members and demonstrated a heterozygous deletion of exon 3 of GH1 in the parents and homozygous wild type in the sibling (Figure 2).

### 4.1 Treatment and Follow‐Up

After complete history taking and physical examination and consideration of the imaging and laboratory assessments [9], IGHD type IA was diagnosed, and the WES report reinforced it; therefore, based on these results, treatment was initiated with a subcutaneous injection of hGH replacement therapy, which responded favorably at first for 6 months with no adverse effects; then effectiveness and response slowed down, probably due to the production of high titers of anti‐GH antibodies. Therefore, hIGF‐I replacement therapy was converted to the only alternative therapeutic approach, but due to the high cost of treatment and the lack of access to IGF‐I in Iran, it was not possible to continue the treatment, and the patient's progress remained at the same stage. With the prescription of 4 months of occupational therapy, the patient could hold his neck at 11 months.

The tooth eruption was late in the follow‐up visit at 24 months old, and the lower canine teeth have not erupted yet. He started speaking at 18 months with simple and elemental words and tried to walk and stand with help, but after several attempts, he stopped trying and still did not walk.

## 5 Discussion

GH‐1 gene is about 65 kb, including five exons and four introns, and is located on the long arm of chromosome 17 (17q22‐24) where a gene cluster consisting of five structural genes (GH1, GH2, CSHP1, CSH1, CSH2) is expressed [10]. Isolated growth hormone deficiency can be familial (four inheritance patterns) or sporadic. Since magnetic resonance imaging reveals only 12%–20% hypothalamic or pituitary abnormalities in these patients, it is considered that many genetic defects may remain undiagnosed, and many sporadic cases may have a genetic defect [11]. In 2009, Alatzoglou et al. conducted a cohort study that reported 7.4% of patients with GHD had mutations in GH1 with a higher prevalence in familial cases (22.7%) than sporadic ones (2.7%) [12]. In a series of 30 IGHD patients in 2012, the deletion size in 83% was about 6.7 kb, and the height of 31% was SDS > −4 [13]. Deletions in the GH‐1 gene cause IGHD IA from 6.5 to 45 kb, representing about 13% of the familial type of IGHD [8]. Inadequate and unequal recombination and crossover during the meiosis of the GH gene cluster are considered the most common reason for the deletions. Other gene defects such as point mutations, heterozygous frameshift mutations, homozygous nonsense mutations, deletions causing stop codons, frameshifts, and/or splicing errors are also mentioned [14, 15, 16, 17, 18].

In 1992, a study reported that the prevalence of the GH1 deletion in children with severe IGHD (height < −4 SD score) was about 9.4%, 13.6%, and 16.6%, respectively, in the North European, Mediterranean, and Turkish [8]. In 1998, Wagner et al. evaluated the absolute frequency of GH‐1 gene mutations in Asia (18.7%), Mediterranean (11.8%), and Northern Europe (8.7%) populations [6].

The demonstrated phenotype is various in the patients with IGHD due to the underlying genetic defect and may pursue with other pituitary hormone deficiencies, considerably genetic disorders in transcription factors responsible for the Hypothalamus Pituitary axis development or in GH secretion pursue with a higher risk of additional pituitary endocrinopathies, which is about 5.5% in childhood‐onset and 35% in adulthood‐onset [19]. Iamo compared two cases of IGHD and Laron syndrome presenting with severe short stature. Besides growth failure, the case with IGHD revealed a doll face, truncal obesity, frontal bossing, and acromicria without psychomotor development defect, whereas the patient with Laron Syndrome presented with a large head, saddle nose, cranium hypoplasia, underdeveloped mandible, protruding forehead, and a micropenis with high GH levels [20]. Keselman et al. also reported a patient with IGHD presented with a doll face, frontal bossing, truncal obesity, and acromicria in physical examination [21]. Complete absence of endogenous GH production leads to the release of anti‐GH antibodies after GH replacement therapy and prevents the growth response expected from GH replacement therapy, whereas individuals with heterozygous forms of genes whose other GH‐1 allele is not deleted and remains are usually immune tolerant. The first study about IGHD IA was published in 1970 by Ruth Illig, reporting adults with short stature, growth delay, and dwarfism due to IGHD and also demonstrated the high incidence of antibodies after HGH treatment [22]. Early prescription of rhGH has demonstrated favorable results. The preferred treatment for these patients includes rhIGF‐I alone or rhIGF‐I/rhIGFBP‐3 complex [23]. The lack of growth hormone in adults and, as a result, severe deficiency of IGF1 may be the basis of the current metabolic disorders, reduced quality of life, and musculoskeletal diseases [24].

Despite the increasing number of genes related to IGHD, defects in the known genes are found in few cases. Therefore, considering newly discovered mutations can facilitate diagnosis and treatment. therefore, considering novel mutations is required to prevent misdiagnosis, consider timely and appropriate management of the current and long‐term consequences of the defect, such as additional deficiencies, and also long‐term follow‐up considerations [25]. Hence, we report a 16‐month‐old male infant with a homozygous large deletion of approximately 1.6 kb spanning the GH1 gene, containing exons 1–4 with an autosomal recessive inheritance and a heterozygous deletion of exon 3 of GH1 in the parents and homozygous wild‐type in the sibling. The patient initially presented with growth delay (‐4SD). The initial treatment was hGH replacement therapy, which responded well in the first 6 months; then effectiveness and response slowed down. Long‐term follow‐up is needed due to the antibody production.

## 6 Conclusion

This report aimed to reveal a novel homozygous large deletion of approximately 1.6 kb spanning the GH1 gene in a male patient who achieved the criteria for severe IGHD, presented with growth retardation (−4SD) at 4 months old, and due to novel mutations considered as the etiology, antibody production interfering with treatment, and various phenotypes and responses to treatment, more research is needed to improve the prognosis, management, quality of life, and provide methods with lower costs. Furthermore, the clinicians should be aware of the progression to the additional pituitary hormone deficiencies with thyroid and adrenal function tests.

## Author Contributions

**Shahab Noorian:** conceptualization, data curation, investigation, methodology, project administration, supervision, validation, visualization, writing – review and editing. **Hedieh Soltani:** investigation, project administration, visualization, writing – original draft, writing – review and editing. **Fatemeh Aghamahdi:** data curation, investigation, supervision, writing – review and editing. **Shahram Savad:** investigation, methodology, writing – review and editing. **Mahnaz Seifi Alan:** investigation, methodology, writing – original draft, writing – review and editing.

## Ethics Statement

The authors have nothing to report.

## Consent

Written informed consent to publish was obtained from the patient's parents.

## Conflicts of Interest

The authors declare no conflicts of interest.

## Data Availability

The datasets used and/or analyzed during the current study are available from the corresponding author on request, with the approval of the patients' legal guardians.
